# Genome-wide association study reveals the genetic determinism of growth traits in a Gushi-Anka F_2_ chicken population

**DOI:** 10.1038/s41437-020-00365-x

**Published:** 2020-09-28

**Authors:** Yanhua Zhang, Yuzhe Wang, Yiyi Li, Junfeng Wu, Xinlei Wang, Cheng Bian, Yadong Tian, Guirong Sun, Ruili Han, Xiaojun Liu, Ruirui Jiang, Yanbin Wang, Guoxi Li, Wenting Li, Xiaoxiang Hu, Xiangtao Kang

**Affiliations:** 1 College of Animal Science and Veterinary Medicine, Henan Agricultural University, Zhengzhou, 450046 China; 2 College of Animal Science and Technology, China Agricultural University, Beijing, 100193 China; 3 State Key Laboratory of Agrobiotechnology, College of Biological Sciences, China Agricultural University, Beijing, 100193 China; 4 Henan Innovative Engineering Research Center of Poultry Germplasm Resource, Henan Agricultural University, Zhengzhou, 450046 China

**Keywords:** Genome-wide association studies, Genetic linkage study, Development, DNA sequencing, Animal breeding

## Abstract

Chicken growth traits are economically important, but the relevant genetic mechanisms have not yet been elucidated. Herein, we performed a genome-wide association study to identify the variants associated with growth traits. In total, 860 chickens from a Gushi-Anka F_2_ resource population were phenotyped for 68 growth and carcass traits, and 768 samples were genotyped based on the genotyping-by-sequencing (GBS) method. Finally, 734 chickens and 321,314 SNPs remained after quality control and removal of the sex chromosomes, and these data were used to carry out a GWAS analysis. A total of 470 significant single-nucleotide polymorphisms (SNPs) for 43 of the 68 traits were detected and mapped on chromosomes (Chr) 1–6, -9, -10, -16, -18, -23, and -27. Of these, the significant SNPs in Chr1, -4, and -27 were found to be associated with more than 10 traits. Multiple traits shared significant SNPs, indicating that the same mutation in the region might have a large effect on multiple growth or carcass traits. Haplotype analysis revealed that SNPs within the candidate region of Chr1 presented a mosaic pattern. The significant SNPs and pathway enrichment analysis revealed that the *MLNR*, *MED4*, *CAB39L*, *LDB2*, and *IGF2BP1* genes could be putative candidate genes for growth and carcass traits. The findings of this study improve our understanding of the genetic mechanisms regulating chicken growth and carcass traits and provide a theoretical basis for chicken breeding programs.

## Introduction

Maynard Smith and Haigh (1974) demonstrated that the occurrence and fixation of a selectively favorable mutation in a population will alter the frequencies of closely linked alleles. The frequency of alleles present in the chromosome where the original mutation occurs will increase, whereas the frequency of other alleles will decrease. We call this the “hitch-hiking effect” because an allele can increase in frequency from selection acting on an adjacent allele (Smith and Haigh 1974). Linkage disequilibrium (LD)between quantitative trait loci (QTL) or markers plays a central role in gene localization (Pritchard and Przeworski 2001). Current QTL mapping methods, such as linkage analysis (LA) and genome-wide association study (GWAS), are based on the LD in the flanking region of one allele to be selected. In addition, polygenic adaptation is a process in which selection leads to a new or optimum phenotype by changing the frequencies of modest alleles. Polygenic adaptation can accelerate the genomic response to environmental changes, however, no selective signatures were produced (Pritchard et al. 2010). A range of adaptive events led by polygenic adaptation are not easily detected by conventional methods (Pritchard and Anna 2010).

Growth traits play an important role in animal production and breeding programs. Growth traits are quantitative traits that are controlled by multiple genes, and it is difficult to make breakthrough progress with conventional breeding methods. Kemper, Visscher 2012 reviewed the literature related to the genetic basis of body size and emphasized the complexity of the genetic structure of body size in species with contributions from many loci with large, intermediate, and small individual effects. The genetic basis of body size in species, such as cattle (Saatchi et al. 2014), mice (William et al. 2006), chickens (Brandt et al. 2017), and pigs (Yoo et al. 2014), is commonly polygenic. Previous studies identified many QTLs associated with growth traits and carcass traits (Ankra-Badu et al. 2010a; Baron et al. 2015; Demeure et al. 2013; Huang et al. 2018). The chicken QTL database (Hu et al. 2013) contains 3692 QTLs associated with growth traits, covering nearly the entire genome except Chr 30–33 and ChrW. Most of the reported QTLs were mapped by low-density microsatellite markers that were inadequate for fine mapping. However, using high-density whole-genome single-nucleotide polymorphism (SNP) markers could greatly improve the mapping accuracy while implementing GWAS correlation analysis. Here, we performed a GWAS using 321,314 SNPs for chicken growth traits and carcass traits in 734 chickens from a Gushi-Anka F_2_ resource population. The results deepen our understanding of the genetic architecture of growth and carcass traits in chickens.

## Materials and methods

### Ethics statement

The animal experiments in this study were approved by the Institutional Animal Care and Use Committee (IACUC) of Henan Agricultural University under approval number 17-0118. All experiments strictly followed the guidelines of this committee.

### Experimental population and phenotyping

The Gushi-Anka F_2_ chicken resource population used in this study was established by the Henan Innovative Engineering Research Center of Poultry Germplasm Resource for genomic studies. The F_2_ resource population consisted of four cross-bred families (Anka-cocks mated with Gushi-hens) and three reciprocal families (Gushi-cocks mated with Anka-hens) as described previously (Han et al. 2012), and a total of 860 F_2_ chickens were finally generated. All chickens had free access to feed and water and were managed under the same environment.

Phenotypes, including growth traits and carcass traits, were measured for all 860 chickens. Growth traits consisted of body weight (BW) and body size. BW was recorded individually every 2 weeks from birth to 12 weeks (BW0, BW2, BW4, BW6, BW8, BW10, and BW12). Body size was measured at 4, 8, and 12 weeks and included shank length (SL), shank girth (SG), chest depth (CD), chest width (CW), breast bone length (BBL), pectus angle (PA), body slanting length (BSL) and pelvis breadth (PB). Carcass traits were collected after 860 F_2_ chickens were slaughtered at the age of 12 weeks. Seventeen carcass traits were measured, including carcass weight (CWe), semi-evisceration weight (SEW), evisceration weight (EW), liver weight (LW1), heart weight (HW), gizzard weight (GW), spleen weight (SW), pancreas weight (PW), head weight (HW1), claw weight (CW1), double pinion weight (DPW), breast muscle weight (BMW), leg muscle weight (LMW), leg weight (LW), fat bandwidth (FBW), skin fat thickness (SFT), and abdominal fat weight (AFW). Moreover, the percentage traits were also calculated. BMR, LMR, LR, and AFR were the ratios of BMW, LMW, LW, and AFW to EW, and the other ratios for each carcass trait were calculated by dividing their weight by BW12, for example, CWR = CWe/BW12. The measurement methods have been previously detailed by Han et al. (2011) and Li et al. (2019). The phenotypes that did not follow a normal distribution (shown in Table S1 with an asymptotic *P* < 0.05) were ranked by normal scores with the Tukey method, and then transformed data were used in the following genetic analyses.

### Genotyping, imputation, and quality control

Individual genomic DNA was isolated from blood samples by the Qiagen DNeasy Blood and Tissue Kit (Qiagen, Hilden, Germany) according to the manufacturer’s instructions. The DNA quality and concentration satisfied the requirements for library construction of double-digest genotyping-by-sequencing (ddGBS) (Wang et al. 2017). In total, 768 qualified genomic DNA samples were genotyped using ddGBS. Then, libraries were sequenced on an Illumina HiSeq X Ten platform (PE150). Read pairs were excluded when either read of a pair satisfied any of the following criteria: contained adaptor sequences, >50% low-quality bases, or >5% N bases. Filtered paired reads were aligned to the chicken reference genome Gallus_gallus-5.0 (released 2015) using Bowtie2 (version 2.3.0) (Langmead and Salzberg 2012).

SNPs were identified using the TASSEL GBS analysis pipeline (version 5.2.31) (Glaubitz et al. 2014; Van Tassell et al. 2008). Quality control was carried out using VCFtools (version 0.1.13) (Petr et al. 2011). SNPs were retained if they satisfied the following criteria: minor allele frequency >0.05; genotypes with a quality above 98 (minGQ ≥98) and depth ≥5; two alleles; consistent with Hardy–Weinberg equilibrium; and max missing rate <0.4. Samples with both side call rates <0.3 were excluded. Missing genotypes were imputed according to the information of the remaining SNPs with Beagle4.0 software (Browning and Browning 2013). SNPs located in sex chromosomes (ChrZ and ChrW) were removed before GWAS analysis.

### GWAS

Population structure is a major origin of confounding effects in genetic analysis. Prior to GWAS analysis, principal component analysis (PCA) was implemented using GCTA (Jian et al. 2011) software to assess the population structure. Considering that the high LD between adjacent SNPs may bias the PCA results, all autosomal SNPs were pruned to obtain independent SNPs. Then, the top two principal components (PCs) were calculated and used as covariates in the mixed model. In addition, the genomic relationship matrix was constructed with SNPs using GCTA software and used as a random effect in the mixed model.

GWAS analysis was carried out for growth traits and carcass traits using a mixed linear model (MLM) in the GCTA program.

The following MLM was used:

$${\mathit{y}} = {\mathit{W}}\alpha + \beta {\mathit{x}} + u + e$$

where *y* is the phenotypic values for each trait; *W* is a matrix of covariates (fixed effects) that control the population structure (top two PCs), sex and genotyping batch effect; in particular, BW0 is also considered as a covariate in all BW traits; *α* is a vector of corresponding coefficients including the intercept; *β* is the SNP effect and *x* is a vector of SNP genotypes; *u* is a vector of random effects with a covariance structure that follows a normal distribution as *u* ~ *N* (0, KV_g_), where *K* is a known genetic relationship matrix; and *e* is a vector of random errors.

The genome-wide significance *P* value threshold was calculated using Bonferroni correction with an effective number of independent tests. We calculated the number of genome-wide independent markers using PLINK (Purcell et al. 2007) -indep-pairwise, with a window size of 25 SNPs, a step of five SNPs, and an *r*^2^ threshold of 0.1. The significance level was set as 1.90E-06 (0.05/26 352; −log_10_(*P*) > 5.72) based on the 26,352 independent markers. Manhattan and Q-Q plots were derived from the GWAS results using the CMplot package (https://github.com/YinLiLin/R-CMplot) within the R software (http://www.r-project.org/).

### Post-GWAS analysis

The Ensembl genome database and SNPEff (version 4.1) program (Cingolani et al. 2012) were used to obtain information about SNPs and perform relevant gene annotation of the GWAS results. Additionally, we utilized the Circos (http://circos.ca/) software package (Alexander et al. 2013) to visualize the distribution of chromosome length, SNPs, GC islands, and repeat regions in the chicken genome. The GOseq R package [58] was used to identify significantly enriched GO terms with corrected *P* values < 0.05 for 277 candidate genes. We used the clusterProfiler R package to test the statistical enrichment of the 277 candidate genes in KEGG pathways (Mao et al. 2005) to identify enriched pathways. Pathways with *P* values < 0.05 were identified as significantly enriched in the candidate genes.

## Results

### Phenotype and genotype statistics

**Fig. 1:**
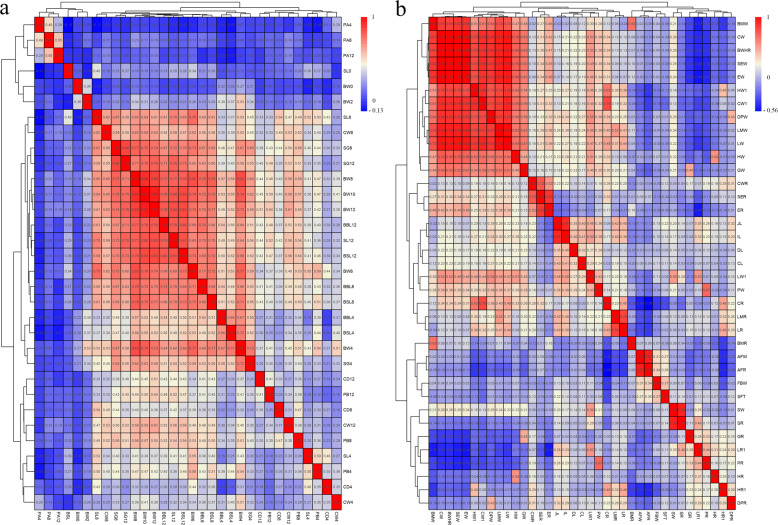
Pearson correlation between phenotypes. The Pearson correlation coefficients of pairs of traits were calculated, and the traits were clustered based on the correlation coefficients. The pairwise Pearson’s correlation of 32 growth traits (including birth body weight (BW)) and 37 carcass traits are shown in Fig. 1a and Fig. 1b, respectively. The colors (numbers) represent the pairwise correlation coefficients of the serum biochemical indicators. Red indicates a positive correlation, and blue indicates a negative correlation. Figure 1a shows growth traits including BW at 0/2/4/6/8/10/12 weeks (BW0/2/4/6/8/10/12), birth shank length (SL0), shank length at 4/8/12 weeks (SL4/8/12), shank girth at 4/8/12 weeks (SG4/8/12), chest depth at 4/8/12 weeks (CD4/8/12), chest width at 4/8/12 weeks (CW4/8/12), breast bone length at 4/8/12 weeks (BBL4/8/12), pectus angle at 4/8/12 weeks (PA4/8/12), body slanting length at 4/8/12 weeks (BSL4/8/12), and pelvis breadth at 4/8/12 weeks (PB4/8/12). Figure 1b shows carcass traits at 12 weeks including carcass weight (CWe), semi-evisceration weight (SEW), evisceration weight (EW), liver weight (LW1), heart weight (HW), gizzard weight (GW), spleen weight (SW), pancreas weight (PW), head weight (HW1), claw weight (CW1), double pinion weight (DPW), breast muscle weight (BMW), leg muscle weight (LMW), leg weight (LW), fat bandwidth (FBW), skin fat thickness (SFT) and abdominal fat weight (AFW), BW of hair removed (BWHR), duodenum length (DL), jejunum length (JL), ileum length (IL), cecum length (CL), ratios of CWe/SEW/EW/LW1/HW/GW/SW/PW/HW1/CW1/DPW to BW12 (CWR/SER/ER/LR1/HR/GR/SR/PR/HR1/CR/DPR), and ratios of AFW/BMW/LMW/LW to EW (AFR/BMR/LMR/LR).

Descriptive statistics for growth and carcass straits of the F_2_ resource population are listed in Table S1. We calculated the pairwise Pearson’s correlation of 32 growth traits (including birth BW) and 37 carcass traits. As shown in Fig. 1a for growth traits, BW, and body size (BBL, SL, BSL, and CW) at the mid- and late-growth stages were strongly correlated (Pearson’s *r* > 0.69). For example, the pairwise Pearson’s *r* of BW8, BW10 and BW12 was >0.85. In addition, different body size measurements, including SL, CW, SG, BBL, and BSL, showed stronger correlative relationships at the same stage (4 weeks, 8 weeks, and 12 weeks old). However, a relatively weak correlation was observed in both the BW and partial body size traits (PA, CD, and PB) between the early- and late-growth stages. Furthermore, a strong correlation occurred between the CWe trait and parts of the CWe, such as the BMW, LMW, HW1, LW, CW1, EW, and SEW (Fig. 1b), however, a weak correlation occurred between the CWe and the organ weights or fat weights. We classified the traits with high correlation into a single cluster trait.

A total of 7,258 million clean reads and 6,071 million good barcode reads were obtained after 768 samples were sequenced. Both the number of reads and the data quality were satisfactory for subsequent analysis (Table 1). Finally, 734 chickens and 321,314 SNPs remained after quality control and removal of the sex chromosomes. Among all discovered SNPs, 35,677 (~10.59%) were novel to the NCBI chicken dbSNP database. The density, distribution and variant rate of SNPs across chromosomes are listed in Table S2, which indicates that the average SNP density per chromosome was 309 SNPs/Mb. According to the physical distance and recombination distance reported above, the SNP density satisfied the standard for GWAS analysis.

**Table 1:** Statistics of sequenced data from eight different lanes.

Lane	Bases (G)	Total reads	Q30 (%)	Q20 (%)	R1 reads	R1 good barcode reads	R1 good ratio	R2 reads	R2 good barcode reads	R2 good ratio	Filter
L1	121	808 639 426	92	96	404 319 713	295 785 461	0.73	404 319 713	351 873 955	0.87	7
L2	125	832 928 728	93	97	416 464 364	295 646 345	0.71	416 464 364	357 060 282	0.86	5
L3	127	848 358 674	93	97	424 179 337	299 066 848	0.71	424 179 337	366 820 501	0.86	7
L4	144	961 606 222	93	97	480 803 111	404 804 456	0.84	480 803 111	413 407 380	0.86	3
L5	145	968 786 086	93	97	484 393 043	405 210 921	0.84	484 393 043	418 048 747	0.86	3
L6	142	948 387 596	92	97	474 193 798	402 730 494	0.85	474 193 798	418 114 080	0.88	3
L7	141	940 898 906	92	97	470 449 453	409 728 378	0.87	470 449 453	415 260 489	0.88	3
L8	142	948 527 654	92	97	474 263 827	403 069 787	0.85	474 263 827	414 036 702	0.87	3
Total	1 089	7 258 133 292	92.63	96.57	3 629 066 646	2 916 042 690	0.80	3 629 066 646	3 154 622 136	0.87	34

Eight libraries were constructed, with 96 samples per library, and a total of 768 samples were subjected to genotyping. Lanes 1–8 represent eight libraries. Bases refer to the amount of sequencing data. R1 reads and R2 reads refer to the reads for the paired end sequence (PE150). Total reads represents the sum of R1 reads and R2 reads. A good barcode read is a sequence read that matches one of the barcodes used in ddGBS. R1 good barcode reads and R2 good barcode reads refer to the good barcode reads for the paired end sequence (PE150). R1 good ratio = R1 good barcode reads/R1 reads. R2 good ratio = R2 good barcode reads/R2 reads. The filter refers to the number of filtered individuals.

### Population structure

**Fig. 2:**
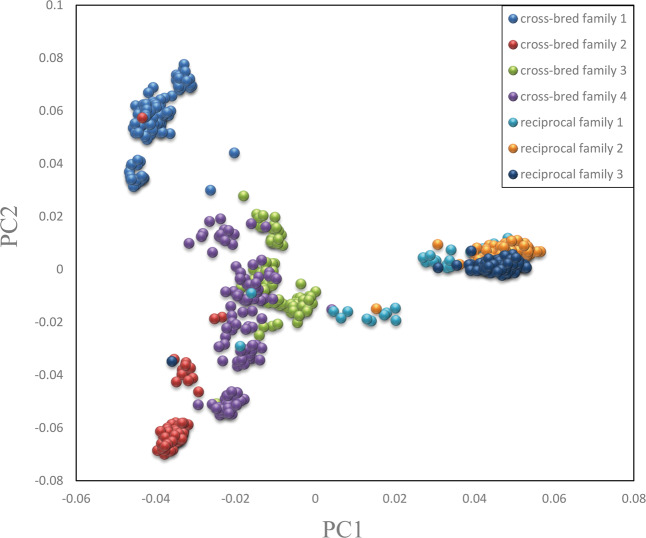
PCA for population structure. Each dot in this figure corresponds to one individual within the families. Dots of different colors represent individuals from different families. Blue, red, green, and purple represent four cross-bred families; sky blue, yellow, and dark blue represent three reciprocal families. PC1 and PC2 explained 18.7% and 11.3% of the variance, respectively.

PCA using the first two principal components (Fig. 2) showed that the cross-bred and reciprocal families formed separate clusters and were separate from each other. Individuals from the four cross-bred families clearly grouped into their respective families. However, three reciprocal families clustered together. The variances explained by PC1 and PC2 were 18.7% and 11.3%, respectively. Therefore, population stratification could be accounted for in the linear mixed model by including the first two PCs as covariates in the GWAS analysis.

### GWAS

**Fig. 3:**
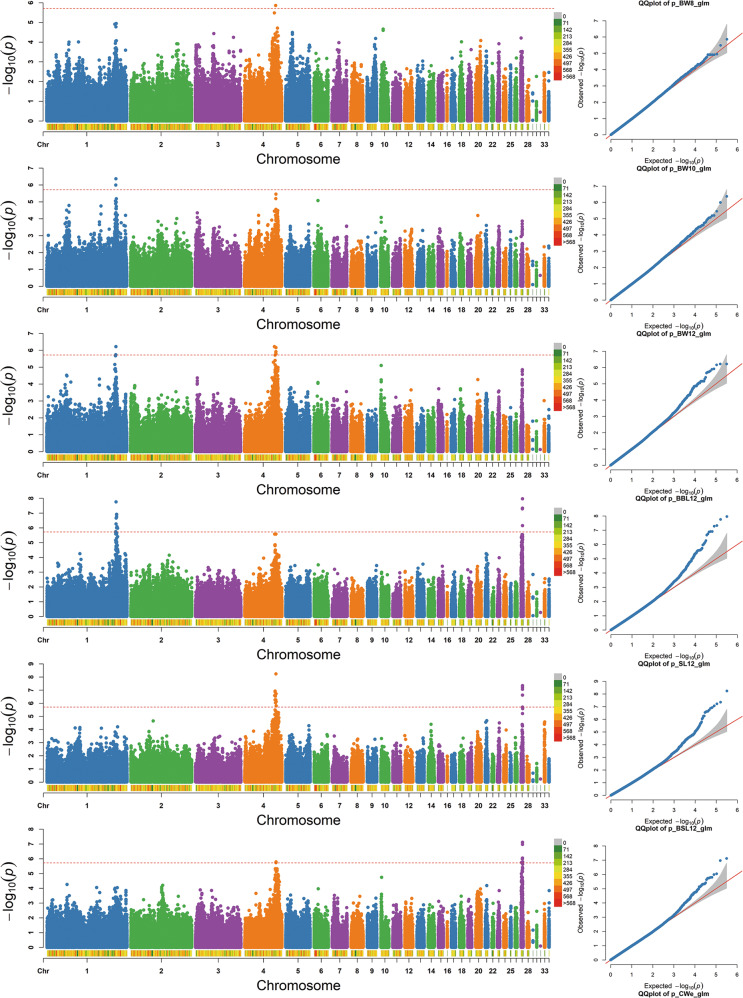

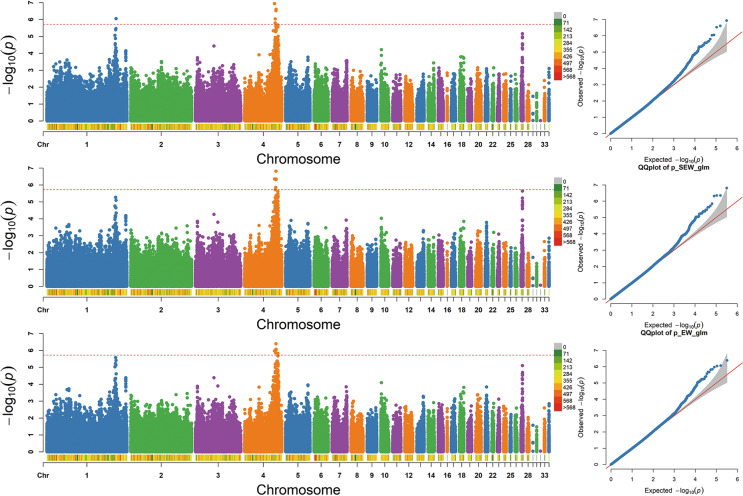
The Manhattan and Q-Q plots for nine traits. The nine traits shown in Fig. 3 were the main focus of our study and were strongly correlated with both growth traits and carcass traits. In addition, the pairwise correlation coefficients for six growth traits (BW8, BW10, BW12, BBL12, SL12, and BSL12) were >0.77, located in the central red block in Fig. 1a. Similarly, strong correlation events were also observed for three carcass traits (CWe, SEW, and EW), of which pairwise correlation coefficients were >0.99, located within the red block at the left top (Fig. 1b). Each dot in this figure corresponds to an SNP within the data set. In each Manhattan plot, the dot color indicates the chromosome on which the SNP is located, the dot position indicates the −log_10_-transformed *P* value of the SNP, the number below represents the chromosome number, the length of the figure above the number represents the length of the chromosome, and the color represents the number of SNPs on the chromosome. The horizontal red dashed line denotes genome-wide significance (−log_10_(*P*) > 5.72). For each Q-Q plot, the *x* axis represents the expected −log_10_-transformed *P* value, the *y* axis shows the observed −log_10_-transformed *P* value, and the red line is the diagonal line.

**Fig. 4:**
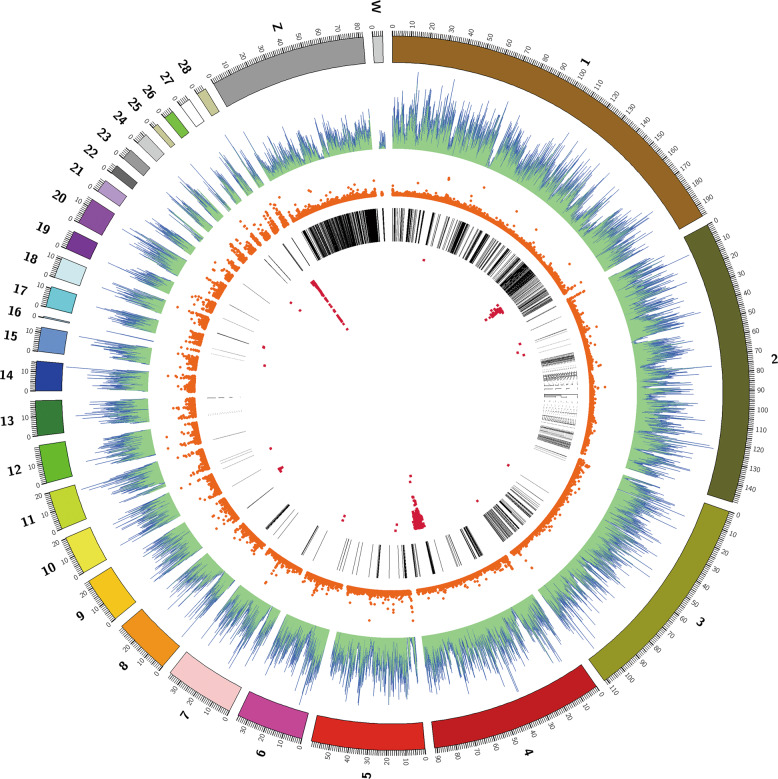
SNPs significantly associated with 43 of the 68 traits at genome-wide significance. The exterior circle represents the lengths of the chromosomes. The four inner circles represent the distribution of SNPs (green), GC islands (orange), repeat regions (black), and whole-genome significant SNPs for growth and carcass traits. The 336,882 SNPs generated by ddGBS are evenly distributed on the chromosome. Each red dot in the inner circle in this figure represents a significant SNP, with a total of 470 SNPs significantly associated with 43 of the 68 growth and carcass traits at genome-wide significance (−log_10_(*P*) > 5.72).

Manhattan and Q-Q plots for 31 growth traits and 37 carcass traits were constructed (Fig. 3 and Fig S1). The single-marker analysis identified 470 SNPs significantly associated with 43 of the 68 traits with genome-wide significance (−log_10_(*P*) > 5.72) (Fig. 4).

#### I. GWAS for growth traits

Of the above 470 SNPs, 103 genome-wide significant SNPs were associated with 18 growth traits and mapped to Chr1, -2, -4, -5, -16, -18, -23, and -27 (Table S3). Based on the SNP annotation, we found that 23 of the significant SNPs were located in intergenic regions, 20 were in introns and 13 were upstream of the coding sequences. Moreover, the significant SNPs associated with BW at different ages (including BW4, BW6, BW8, BW10, and BW12) were all located on Chr1, -4, and -27. Body size traits at 12 weeks old were all significantly associated with SNPs on Chr27. Through a linear regression analysis with the most significant SNP (i.e., Chr27: 3,971,467) and BW traits, we found that this SNP (−log_10_(*P*) = 7.35; intergenic) explained 2.31% of the phenotypic variance. The genomic control inflation factor (*λ*) calculated for all growth traits ranged from 0.97 to 1.00, which indicated acceptably low false positives (Fig. 3 and Fig S1).

#### II. GWAS for carcass traits

Similarly, GWAS analysis was performed for each carcass trait. A total of 367 genome-wide significant SNPs were identified to be associated with 25 carcass traits, which were mapped onto Chr1, -2, -3, -4, -6, -9, -10, -18, and -27 (Table S3). After SNP annotation, 82 significant SNPs were located at intergenic regions, 81 were at introns and 54 were upstream of coding sequences. It was remarkable that the significant SNPs associated with carcass traits with a strong correlation (Fig. 1b) were all located on Chr1, -4, and -27. Except for the BMW, significant SNPs were located on Chr18. The most significant SNP (i.e., Chr27: 3,654,994) were associated with CW1. Linear regression analysis showed that this SNP (−log_10_(*P*) = 14.16; intron) could explain 10.14% of phenotypic variance. The genomic control inflation factor (*λ*) calculated for all growth traits ranged from 0.95 to 1.00, which is similar to the value found for growth traits (Fig. 3 and Fig S1).

### LD analysis

**Fig. 5:**
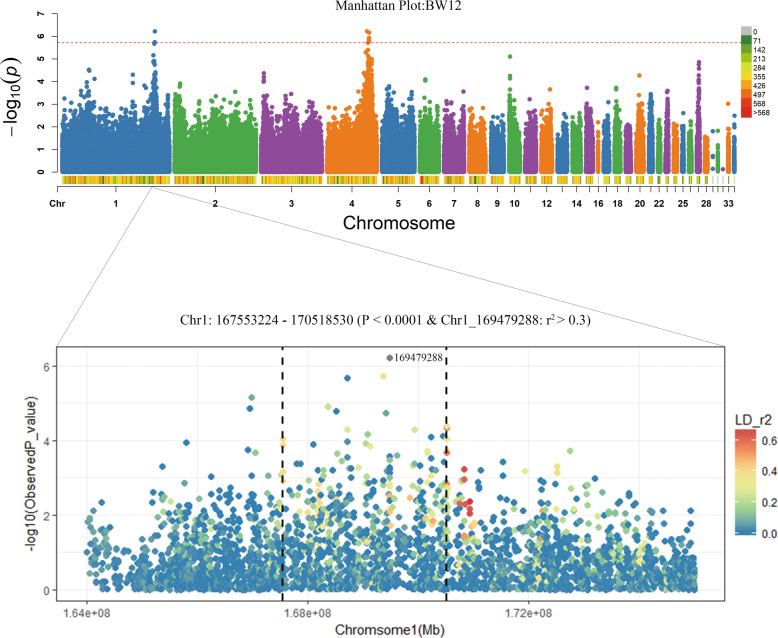
Manhattan plot for BW12. Each dot in this figure corresponds to an SNP within the data set. The graph above is the Manhattan plot for BW12. The horizontal red dashed line denotes genome-wide significance (−log_10_(*P*) > 5.72). The figure below is the enlarged QTL interval diagram. Gray dots represent the most significant SNP loci (Chr1_169,479,288). The color of other SNPs represents the degree of LD with the most significant SNP loci (*r*^2^ value). The remaining SNPs with *r*^2^ > 0.3 and −log_10_(*P*) < 4 genomic regions were obtained within a total range of 3 Mb (Chr1:167,553,224–170,518,530; vertical black dashed line).

The genome-wide significant SNPs were mainly distributed throughout Chr1, Chr4, and Chr27 (Fig. 4). Considering BW at the age of 12 weeks as slaughter weight, we focused on the BW12 trait for further analysis. The Manhattan plot of GWAS for BW12 is shown in Fig. 5. The lead SNPs were located at Chr1:169,479,288 and Chr4: 73,497,549. After retaining the lead SNPs and their neighboring SNPs with *r*^2^ > 0.3 and −log_10_(*P*) < 4, two genomic regions (Chr1:167.5–170.5 Mb and Chr4: 70.9–81.7 Mb) were obtained within a total range of 3 Mb.

**Fig. 6:**
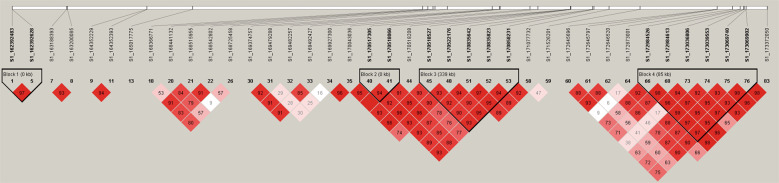
LD blocks with SNPs that affect BW. SNPs on Chr1 satisfying the following criteria were used to construct the haplotype. i. SNP set I, significant SNPs associated with BW at different ages in weeks; ii. SNP set II, significant SNPs associated with traits strongly correlated with BW (Pearson’s *r*^2^ > 0.54); iii. SNP set III, high-LD SNPs with SNP set I, and SNP set II (*r*^2^ > 0.7) and the removal of one of any pair of adjacent SNPs <100 bp apart. Two blocks were obtained with these 39 SNPs (162,392,483–173,372,050), located at 170,518,527–170,858,231 (170.52–170.86 Mb) and 172,984,526–173,069,902 (172.98–173.07 Mb).

We found that significant SNPs on Chr1 had effects on multiple traits, and these SNPs were located in relatively large QTLs, which might affect BW together. Therefore, we screened the SNPs with the following criteria and obtained different SNP data sets. i. SNP set I, significant SNPs associated with BW at the age of different weeks; ii. SNP set II, significant SNPs associated with traits strongly correlated with BW (Pearson’s *r*^2^ > 0.54); iii. SNP set III, high-LD SNPs with SNP set I and SNP set II (*r*^2^ > 0.7) with the removal of one of any pair of adjacent SNPs <100 bp apart. The SNPs obtained from the three datasets are listed in Table S4. However, only two smaller blocks were obtained after haplotype construction with these SNPs (Fig. 6). LD analysis revealed that the SNPs within the two blocks were relatively complex. For the block located at 172.98–173.07 Mb, there were nine SNPs with high linkage with all other SNPs. For the block at 170.52–170.86 Mb, no obvious linkage phase was observed. Both blocks presented a mosaic pattern (Fig S2), which made it difficult to identify causal SNPs. Significant SNPs associated with strong correlation traits within the interval were not highly interlocked.

### Large effects of Chr1, -4, and -27 loci on multiple traits

We found several significant SNPs associated with multiple traits (Table S3). The most association events were observed between Chr4: 73,497,549 and 12 traits (BW12, SL12, SG12, CWe, SEW, EW, HW1, CW1, LMW, LW, BWHR, and CR). Moreover, another two significant SNPs with a distance of ~3 Mb within Chr4: 73,497,549 were also observed to be associated with 11 traits. Similarly, Chr1: 169,479,288 and 169,374,666 were associated with seven traits, and Chr27: 3,654,994, together with its neighboring SNPs, was associated with nine traits. Because the main SNPs were shared by multiple traits, a single mutation in the region might have a large effect on multiple growth and carcass traits.

### Candidate genes

**Fig. 7:**
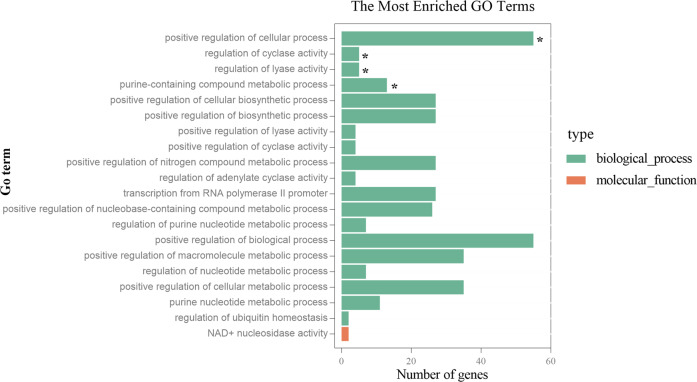
GO enrichment analysis for 277 candidate genes. The *x* axis indicates the number of genes for each GO term; the *y* axis corresponds to the GO terms. The positive regulation of cellular process, the regulation of cyclase activity, the regulation of lyase activity and the purine-containing compound metabolic process were significantly enriched.

**Fig. 8:**
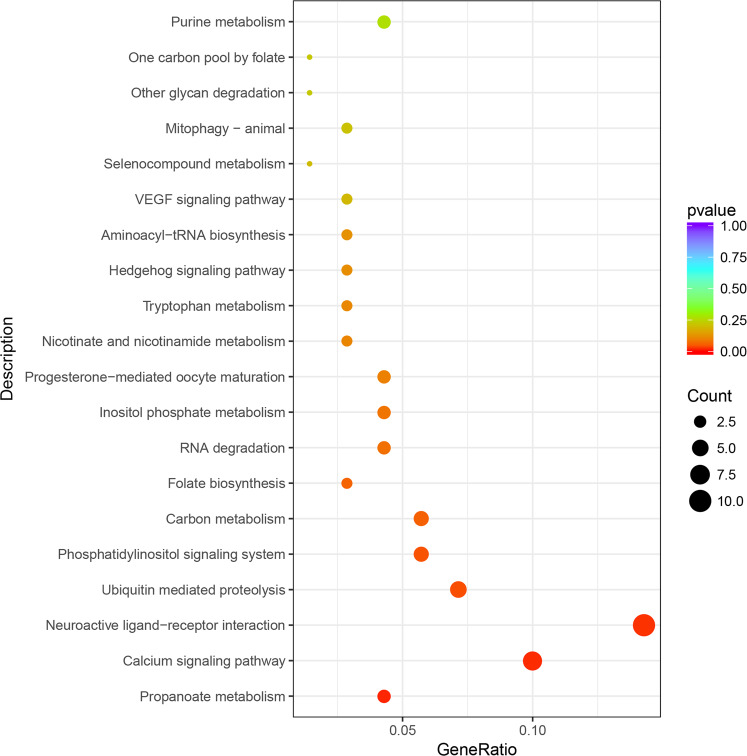
KEGG pathway enrichment analysis for 277 candidate genes. The *x* axis shows the gene ratio; the *y* axis corresponds to KEGG pathways. The dot color represents the *P* value, and the dot size represents the number of genes enriched in the reference pathway. The propanoate metabolism pathway, calcium signaling pathway, neuroactive ligand–receptor interaction pathway, ubiquitin-mediated proteolysis pathway and phosphatidylinositol signaling system pathway were significantly enriched.

Annotation of each significant locus revealed putative candidate genes. Moreover, the genes located within the high-LD region (*r*^2^ > 0.3 and −log_10_*P* < 4) neighboring the significant locus also remained. Finally, a total of 277 unique putative candidate genes were used for enrichment analysis (Table S5). Four GO terms were significantly enriched, including the positive regulation of cellular process, the regulation of cyclase activity, the regulation of lyase activity and the purine-containing compound metabolic process (Fig. 7 and Table S6). Five KEGG pathways were significantly enriched, including the propanoate metabolism pathway, the calcium signaling pathway, the neuroactive ligand–receptor interaction pathway, the ubiquitin-mediated proteolysis pathway and the phosphatidylinositol signaling system pathway (Fig. 8 and Table S7). Among these, the neuroactive ligand–receptor interaction pathway contains a large number of growth-related hormone receptors (Fig. S3), among which the Motilin Receptor (*MLNR*) gene was enriched.

## Discussion

A large number of GWAS have shown that most comprehensive traits and diseases are controlled by multiple genes, and the effect of a single gene is relatively small. For example, a joint analysis of 183,727 people identified 180 height-related loci, which explained a total of only 12% of the phenotypic variations (Hana et al. 2010). In livestock and poultry studies, the genetic determinism of only a handful of traits has been verified to be controlled by the major genes. Generally, most traits have not yet been fine-mapped, although thousands of QTLs affecting various traits are listed in the animal QTL database (https://www.animalgenome.org/cgi-bin/QTLdb/index). Chicken growth traits are a class of complex traits with important economic impacts. Analyzing the genetic basis not only served as a key link for chicken breeding programs but also enhanced our understanding of the genetic pattern of QTLs.

The BW trait is an important variable to evaluate the profitability of broiler production. Changes in BW are often accompanied by the development of body size, and most body size traits show significant positive correlations with BW. Therefore, body size traits can also be used as a reference index for BW (Ajayi et al. 2008; Gogoi and Mishra 2013; Yang et al. 2007). The results of our study indicated that BW traits are strongly correlated with carcass traits (CWe, SEW, and EW) and body size traits (BBL12, SL12, and BSL12). Interestingly, this study found that most of the growth traits, such as chicken BW and body size at mid- and late-growth stages, were significantly associated with the same three QTLs on Chr1, -4, and -27, and these results are identical to those of previous studies (Li et al. 2018; Nassar et al. 2012; Yuan et al. 2018).

Since 2002, eight independent research teams, using LA, association analysis, selection evolution analysis and molecular validation, coincidentally found QTLs on the end of Chr1 with significant effects on growth traits in different chicken breeds and populations (Abdalhag et al. 2015; Besnier et al. 2011; Gao et al. 2009; Hee-Bok et al. 2006; Jia et al. 2016; Kerje et al. 2003; Liang et al. 2012; Liu et al. 2007; Liu et al. 2008; Pettersson et al. 2013; Podisi et al. 2013; Sheng et al. 2013; Uemoto et al. 2009; Zhang et al. 2011). Researchers at South China Agricultural University identified a narrow 1.5 Mb region (Chr1: 173.5–175.0 Mb) of the chicken genome to be strongly associated with chicken growth traits (Liang et al. 2012). Then, a 54 bp insertion event of the miR-16 gene was identified by expression analysis of all genes in the region, cell validation and population verification, and it was found to promote the growth of chicken fibroblasts; therefore, this mutation was identified as the causal mutation within this region (Liang et al. 2012). The Northeast Agricultural University resource population was used to fine-map the QTLs affecting BW at 9–12 weeks. The genomic region, 169.8–175.3 Mb on Chr1, was identified (Liu et al. 2008), and the *RB1* gene within this region was determined to be the major gene by haplotype association analysis (Zhang et al. 2011). China Agricultural University performed a GWAS in an F_2_ intercross by 60 K chip and mapped the major QTLs of BW to the end of Chr1 (173.7 Mb) (Sheng et al. 2013). In our study, QTLs affecting BW4, BW10, and BW12 were also identified at 169.4 Mb on Chr1. It is worth noting that the digestive organ (stomach and pancreas) weights are also significantly associated with loci on Chr1. We found many growth-related genes after listing the genes nearest to significant SNPs. For example, the Motilin Receptor (MLNR) gene, which is a star gene involved in the regulation of growth hormone release, is enriched in the neuroactive ligand–receptor interaction pathway. Mediator Complex Subunit 4 (*MED4*, i.e., vitamin D receptor interacting protein) at Chr1 functions in the regulation of vitamin D metabolism by binding to the vitamin D receptor, further affecting the development and maintenance of mineral ion homeostasis and skeletal integrity (Sutton and MacDonald 2003). The calcium-binding protein 39-like *(CAB39L*, i.e., *MO25*) gene located at Chr1 catalyzes the process of phosphorylation to activate AMP-activated protein kinase and then regulates food intake (Minokoshi et al. 2004; Proszkowiec-Weglarz et al. 2006). The *MLNR* gene encodes a motilin receptor that promotes the release of growth hormone. Its ligand, Motilin, which acts as a secreted protein, directly participates in the interdigestive migrating motor complex, which manifests in intermittent peristalsis of the gastrointestinal tract during digestion to promote food emptying (Itoh and Sekiguchi 1983). Therefore, we considered that the *MLNR* gene is an important candidate gene for downstream functional verification. The BW and growth rate of chickens depend on the balance between feeding and energy consumption. Therefore, genes in this region may affect two biochemical processes, food intake and energy consumption. The results of both our and multiple previous studies have repeatedly confirmed that QTLs located at the end of Chr1 have significant genetic effects on growth traits (Li et al. 2020).

Owing to limited population recombination events, severe epistatic effect interference, insufficient genetic marker density and small phenotypic variance in a single locus, the progress of fine mapping the end of Chr1 is slow. Since 1957, Virginia Polytechnic Institute and State University has constructed high- and low-growth lines of chickens developed from White Plymouth Rock chickens, which have been widely used in research on growth traits such as chicken BW. The F40 generation of the high- and low-growth lines was used to construct positive and negative F_2_ families, respectively, to map the QTLs affecting AFW, BMW, and shank weight. The results of these traits have been located at the end of Chr1 and Chr3, -4, and -27 (Hee-Bok et al. 2006). In 2011, the confidence interval of the major QTL of Chr1 was reduced to 1/3 of the F_2_ generation interval based on deep hybridization of the high- and low-growth lines (Besnier et al. 2011). Using the F9 generation of the AIL family, it was found that the beneficial alleles at the linked fine-mapped loci within this group were located on different haplotypes at the onset of selection, and there were many mosaic points, making it difficult to perform fine mapping (Brandt et al. 2017). This also explains the states that adjacent SNPs in the genomic region of our population are not linked. Both blocks located at the end of Chr1 presented as a mosaic pattern indicating that the underlying genetic mechanisms of growth traits are controlled by micro effect polygenes, and it is difficult to screen candidate genes based on linkage.

Sewalem et al. used an F_2_ chicken population from a cross of a broiler sire-line and an egg laying (White Leghorn) line to identify QTLs on Chr1, -2, -4, -7, -8, and -13 that affected BW, and Chr4 had the largest single additive effect (Sewalem et al. 2002). Researchers identified QTLs on Chr4 that affected growth performance in different varieties and populations (Brandt et al. 2017; Jin et al. 2015; Li et al. 2018), such as a White Leghorn × Rhode Island Red cross (Sasaki et al. 2004), a Silky Fowl × White Plymouth Rock cross (Gu et al. 2011), Beijing-You chickens (Liu et al. 2013), and a population of purebred white layers (WLA) with White Leghorn origin (Lyu et al. 2018). In this study, 17 phenotypes, including BW8, BW12, SL12, CWe and the weight of several internal organs, were mapped on Chr4, indicating that genes on Chr4 may have large effects on BW. For example, the LIM domain-binding 2 (*LDB2*) gene located at Chr4 has been reported many times to be associated with chicken growth traits (Dan et al. 2012; Gu et al. 2011; Liu et al. 2013; Wang et al. 2019). Interestingly, another three genes located at Chr4 have also been identified, including *LCOLR*, *NCAPG*, and *FAM184B*. No evidence has shown that they are related to growth traits in chickens, but they have been confirmed to be “domestication genes” in pigs (Rubin et al. 2012).

Park et al. found that Chr27 harbored a highly significant QTL for shank weight but no significant effect on growth (Hee-Bok et al. 2006). However, evidence from another study reported that the BW trait was mapped to Chr27, and the haplotype identified on Chr27 had a significantly positive effect on BW4-8 (Yuan et al. 2018). In addition, the insulin-like growth factor 2 mRNA-binding protein 1 (*IGF2BP1*) gene located at Chr27 has been demonstrated to be the major gene for growth traits in ducks (Zhou et al. 2018). In our study, significant SNPs on Chr27 were associated with BW at 6 weeks and some other growth and carcass traits. For example, traits related to body size, such as BBL12, SL12, and BSL12, have also been mapped to ~3.6 Mb on Chr27. Likewise, China Agricultural University also mapped the major gene of SL to the middle of Chr27 (Sheng et al. 2013). In addition, several studies reported that both the BW and BL traits were mapped to Chr27, indicating the common effect of these loci on growth and carcass traits (Ankra-Badu et al. 2010b; Nassar et al. 2012; Nassar et al. 2015).

Growth is a complex trait and combined process that is influenced by food intake, appetite, energy composition, nutrient distribution, physical activity, metabolic rate, etc. Therefore, the genetic basis of the growth trait might be affected by many loci, each of which only explained a small variation of the trait. The results of selective-sweep mapping suggested that >100 loci might contribute to the selection response (Johansson et al. 2010; Pettersson et al. 2013). Our study conducted GWAS with a resource population for all growth and carcass traits, including BW, body size, CWe and carcass size, and macroscopically detected all potentially significant loci associated with these phenotypes. The results systematically revealed that most growth traits were mapped to Chr1, -4, and -27, and the mosaic inheritance pattern on Chr1 explained the complexity of fine mapping of Chr1. Our research has provided an in-depth understanding of the genetic architecture of complex traits that will be important for further advances in animal breeding.

## Conclusion

This study provided systematic phenotypic measurements and genetic mapping of growth and carcass traits in 734 chickens from a Gushi-Anka F2 population based on ddGBS sequencing data. A total of 470 significant SNPs for growth and carcass traits were detected and mapped on Chr1-6, -9, -10, -16, -18, -23, and -27, including new loci and genes. Remarkable effects of QTLs on Chr1, -4, and -27 were detected. The significant SNPs associated with multiple traits were all located on Chr1, -4, and -27. Haplotype analysis of the QTL on Chr1 showed a mosaic inheritance pattern. Significant SNPs and pathway enrichment revealed that the MLNR, MED4, CAB39L, LDB2, and IGF2BP1 genes could be putative candidate genes for further study.

## Supplementary information

Supplementary Materials

Supplementary Table S3

Supplementary Table S4

Supplementary Table S5

Supplementary Table S6

Supplementary Table S7

## Data Availability

All raw sequence data have been deposited in the NCBI Sequence Read Archive database with the accession number SRR12532401–SRR12532408 (BioProject number PRJNA659316).
