# Supplementary material for: Genome-wide association study reveals the genetic determinism of growth traits in a Gushi-Anka F2 chicken population
Source: Heredity (Edinb). 2020 Sep 28;126(2):293–307. doi: 10.1038/s41437-020-00365-x (PMC8026619; doi:10.1038/s41437-020-00365-x)
Supplement: Supplementary file 1 — Supplementary Materials [file 41437_2020_365_MOESM1_ESM.pdf]

Supplementary Material

Genome-wide association study reveals the genetic determinism of growth traits in a Gushi-Anka F_2 chicken population

Supplementary tables and figures

Table S1A. Descriptive statistics for the growth traits.

Growth traits	Units	No. of records	Min	Max	Mean	SD	Coefficient of variation	Kolmogorov-Smirnov Z	asymptotic-P
BW2	g	671	68.10	185.50	122.55	18.37	14.99	0.66	0.77
BW4	g	675	157.50	464.00	321.80	48.46	15.06	1.16	0.13
BW6	g	701	279.50	915.00	564.71	109.85	19.45	0.82	0.50
BW8	g	673	479.00	1285.00	816.29	144.95	17.76	0.91	0.37
BW10	g	703	447.00	1691.00	1112.17	181.16	16.29	0.54	0.93
BW12	g	715	471.00	2102.00	1351.35	222.88	16.49	0.68	0.74
BSL	cm	730	2.26	3.00	2.58	0.11	4.21	1.13	0.16
SL4	cm	682	3.20	7.25	5.51	0.80	14.55	4.16	0.00
SL8	cm	678	5.60	10.30	7.93	0.88	11.08	1.17	0.13
SL12	cm	707	7.30	12.20	9.39	0.83	8.84	1.94	0.00
SG4	cm	680	2.00	3.50	2.69	0.20	7.54	3.49	0.00
SG8	cm	667	2.90	4.40	3.42	0.27	7.83	2.88	0.00
SG12	cm	700	3.00	4.90	3.85	0.32	8.21	2.46	0.00
CD4	cm	605	3.00	7.00	4.81	0.63	13.05	3.59	0.00
CD8	cm	600	4.00	9.00	6.43	0.79	12.35	2.71	0.00
CD12	cm	647	6.00	10.00	7.93	0.72	9.13	3.18	0.00
CW4	cm	594	2.80	5.50	4.08	0.47	11.55	4.69	0.00
CW8	cm	591	4.00	7.50	5.61	0.52	9.29	3.76	0.00
CW12	cm	638	4.80	10.00	6.40	0.66	10.33	3.57	0.00
BBL4	cm	674	4.60	7.60	6.21	0.50	8.11	1.64	0.01
BBL8	cm	675	6.60	11.10	8.94	0.70	7.80	1.46	0.03
BBL12	cm	722	9.00	13.20	10.99	0.79	7.16	1.61	0.01
PA4	°	376	62.00	90.00	73.18	3.39	4.64	2.80	0.00
PA8	°	369	65.00	86.00	76.73	3.18	4.14	2.24	0.00
PA12	°	416	70.00	92.00	80.47	3.68	4.57	1.75	0.00
BSL4	cm	677	7.50	13.50	11.39	0.79	6.92	1.52	0.02
BSL8	cm	678	12.20	19.60	16.24	1.13	6.95	1.20	0.11
BSL12	cm	725	15.50	23.50	19.76	1.23	6.20	1.08	0.19
PB4	cm	668	4.00	6.50	5.16	0.47	9.19	4.72	0.00
PB8	cm	669	5.00	8.50	6.86	0.67	9.76	3.40	0.00
PB12	cm	716	6.00	11.50	8.67	0.83	9.58	2.56	0.00

Table S1B. Descriptive statistics for the carcass traits.

Carcass traits	Units	No. of records	Min	Max	Mean	SD	Coefficient of variation	Kolmogorov-Smirnov Z	asymptotic-P
CWe	g	723	435.03	1909.48	1210.66	200.95	0.17	0.72	0.68
SEW	g	725	379.69	1706.51	1098.32	189.06	0.17	0.78	0.58
EW	g	726	283.83	1425.78	917.97	163.18	0.18	0.80	0.54
LW1	g	733	16.56	53.98	28.43	5.10	0.18	1.59	0.01
HW	g	733	3.63	10.48	6.56	1.29	0.20	1.45	0.03
GW	g	734	14.35	44.55	27.83	4.92	0.18	1.22	0.10
SW	g	733	0.67	8.62	2.90	1.09	0.38	3.26	0.00
PW	g	728	1.62	5.86	3.34	0.70	0.21	1.73	0.01
HW1	g	731	27.81	68.96	42.68	7.48	0.18	1.98	0.00
CW1	g	732	28.28	103.75	57.86	14.52	0.25	2.13	0.00
DPW	g	734	48.16	209.10	121.37	22.16	0.18	1.07	0.21
AFW	g	458	0.90	61.86	12.44	12.87	1.03	4.13	0.00
BMW	g	727	61.86	250.00	141.02	31.42	0.56	1.07	0.20
LMW	g	718	102.32	316.26	197.39	40.31	0.62	1.12	0.17
LW	g	729	164.84	477.02	296.48	58.55	0.62	1.29	0.07
FBW	cm	733	0.00	1.70	0.73	0.28	0.38	1.46	0.03
SFT	cm	733	0.06	1.55	0.46	0.18	0.39	1.13	0.16
BWHR	g	732	425.60	1882.00	1183.25	198.47	0.17	0.73	0.67
DL	cm	732	13.00	36.00	24.75	3.40	13.74	2.97	0.00
JL	cm	732	27.00	83.00	50.72	9.68	19.09	1.12	0.16
IL	cm	732	28.00	85.00	49.66	8.86	17.85	1.64	0.01
CL	cm	732	10.00	57.00	15.98	2.98	18.68	4.35	0.00
CWR	%	714	76.87	94.83	89.70	2.05	2.29	1.66	0.01
SER	%	711	71.74	86.51	81.36	2.09	2.56	1.03	0.24
ER	%	711	59.54	74.99	67.98	2.15	3.16	0.85	0.47
LR1	%	714	1.39	3.65	2.14	0.37	17.29	1.29	0.07
HR	%	714	0.31	0.98	0.49	0.07	14.98	2.25	0.00
GR	%	715	1.28	3.79	2.09	0.32	15.54	1.76	0.00
SR	%	714	0.08	0.71	0.22	0.08	36.98	3.27	0.00
PR	%	709	0.10	0.50	0.25	0.06	22.46	2.11	0.00
HR1	%	712	2.29	5.50	3.18	0.38	12.06	1.51	0.02
CR	%	713	2.84	6.47	4.27	0.61	14.25	1.24	0.09
DPR	%	715	4.58	13.37	9.02	0.94	10.40	1.76	0.00
AFR	%	452	0.12	6.51	1.51	1.43	94.35	3.69	0.00
BMR	%	718	9.96	20.12	15.26	1.83	11.98	0.99	0.28
LMR	%	710	15.30	25.87	21.37	1.99	9.31	1.12	0.16
LR	%	721	22.87	38.78	32.17	2.47	8	1.12	0.16

"Kolmogorov-Smirnov Z" and "asymptotic P" were the Z value and significance value, respectively, in the results of a normal distribution test (SPSS - nonparametric - 1 sample Kolmogorov-Smirnov test).

Table S2. Distribution of SNPs discovered in 734 individuals across chromosomes.

Chromosome[1]		Genes[2]	Variants	Density	Variants	Novel[6]
Chr.	Size		(SNP)[3]	(SNPs/Mb)[4]	rate(bp/SNP)[5]	
Chr1	196 202 544	2 962	67 301	343	2 915	6 773
Chr2	149 560 735	1 945	52 480	351	2 849	5 612
Chr3	111 302 122	1 715	38 172	343	2 915	3 682
Chr4	91 282 656	1 547	32 011	351	2 851	3 129
Chr5	59 825 302	1 295	21 434	358	2 791	1 945
Chr6	35 467 016	724	13 395	378	2 647	1 202
Chr7	36 946 936	699	12 813	347	2 883	1 360
Chr8	29 963 013	709	9 749	325	3 073	953
Chr9	24 091 566	615	8 641	359	2 788	823
Chr10	20 435 342	583	7 155	350	2 856	831
Chr11	20 218 793	484	6 613	327	3 057	633
Chr12	19 948 154	482	7 798	391	2 558	930
Chr13	18 407 460	498	6 329	344	2 908	585
Chr14	15 595 052	537	5 291	339	2 947	413
Chr15	12 762 846	462	4 316	338	2 957	534
Chr16	652 338	115	173	265	3 770	71
Chr17	10 956 400	390	3 389	309	3 232	307
Chr18	11 053 727	405	3 613	327	3 059	368
Chr19	9 979 828	410	2 936	294	3 399	214
Chr20	14 109 371	472	4 922	349	2 866	547
Chr21	6 862 722	304	2 339	341	2 934	234
Chr22	4 729 743	174	804	170	5 882	90
Chr23	5 786 528	284	1 882	325	3 074	121
Chr24	6 280 547	226	2 272	362	2 764	154
Chr25	2 906 300	312	542	186	5 362	85
Chr26	5 313 770	326	1 393	262	3 814	174
Chr27	5 655 794	430	1 577	279	3 586	301
Chr28	4 974 273	371	1 293	260	3 847	200
Chr30	24 927	6	5	201	4 985	4
Chr31	49 161	5	40	814	1 229	40
Chr32	78 254	17	1	13	78 254	1
Chr33	1 648 031	141	315	191	5 231	103
LGE64	897 576	66	320	357	2 804	153
ChrW	5 160 035	50	367	71	14 060	366
ChrZ	82 310 166	1 137	15 201	185	5 414	2 739
Mean				309	5 787	
Total	1 021 439 028	20 898	336 882			35 677
GWAS_SNPs (Total SNPs except ChrZ and ChrW)			321 314			

[1]Numbers and sizes of chromosomes in *Gallus gallus* 5.0; [2]Numbers of genes on chromosomes in *Gallus gallus* 5.0; [3]Numbers of SNPs on chromosomes as determined by genotyping by sequencing (ddGBS); [4]Density (SNPs/Mb) = no. of variants/size; [5]Variant rate (bp/SNP) = size/no. of variants; [6]SNPs first identified by BLAST analysis with the NCBI chicken dbSNP.

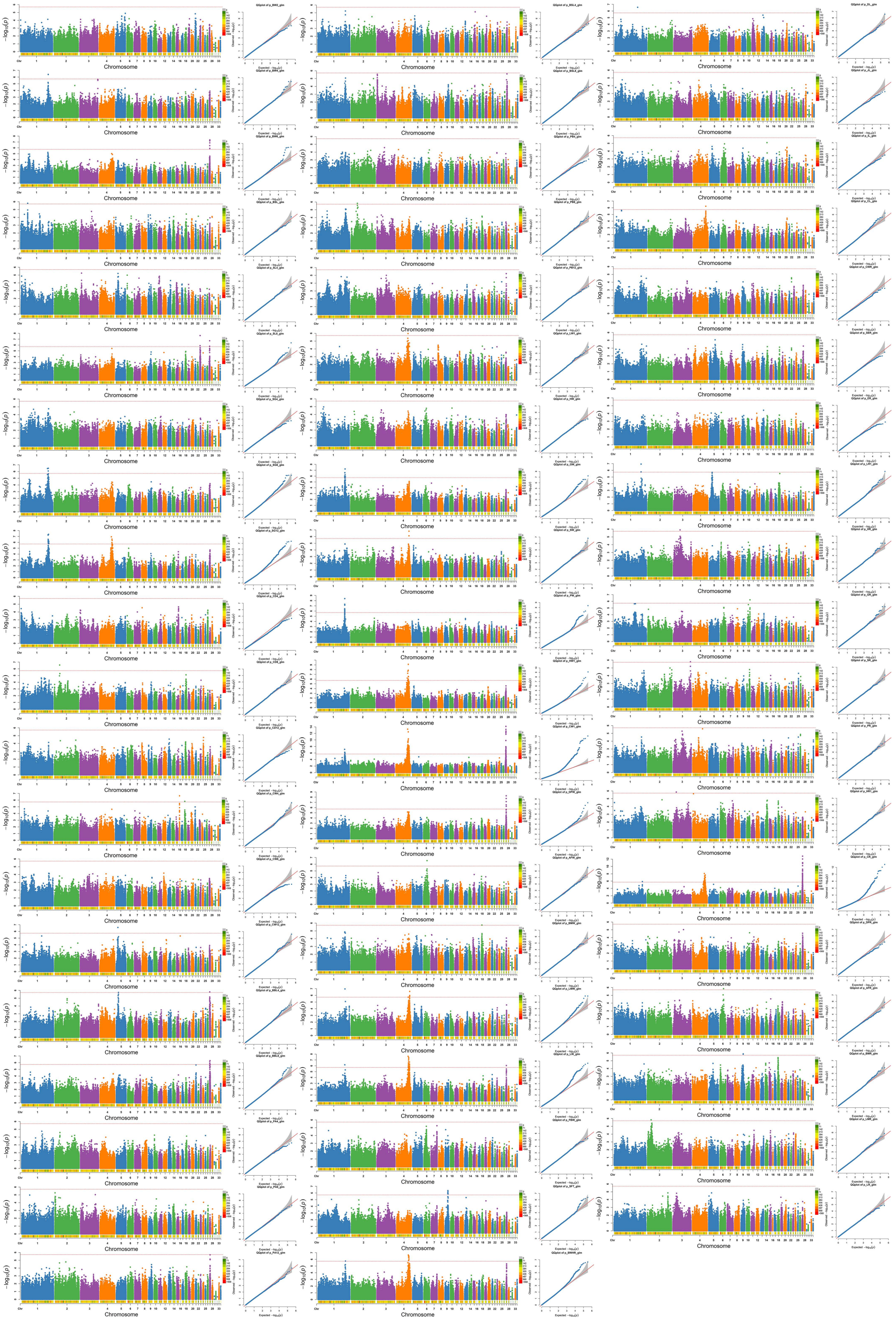

Fig S1. The Manhattan and Q-Q plots for 59 traits. Each dot in this figure corresponds to an SNP within the dataset. The horizontal red dashed line denotes genome-wide significance (-log10(P) > 5.72). The corresponding traits of abbreviations are noted in the explanation of Fig 1.

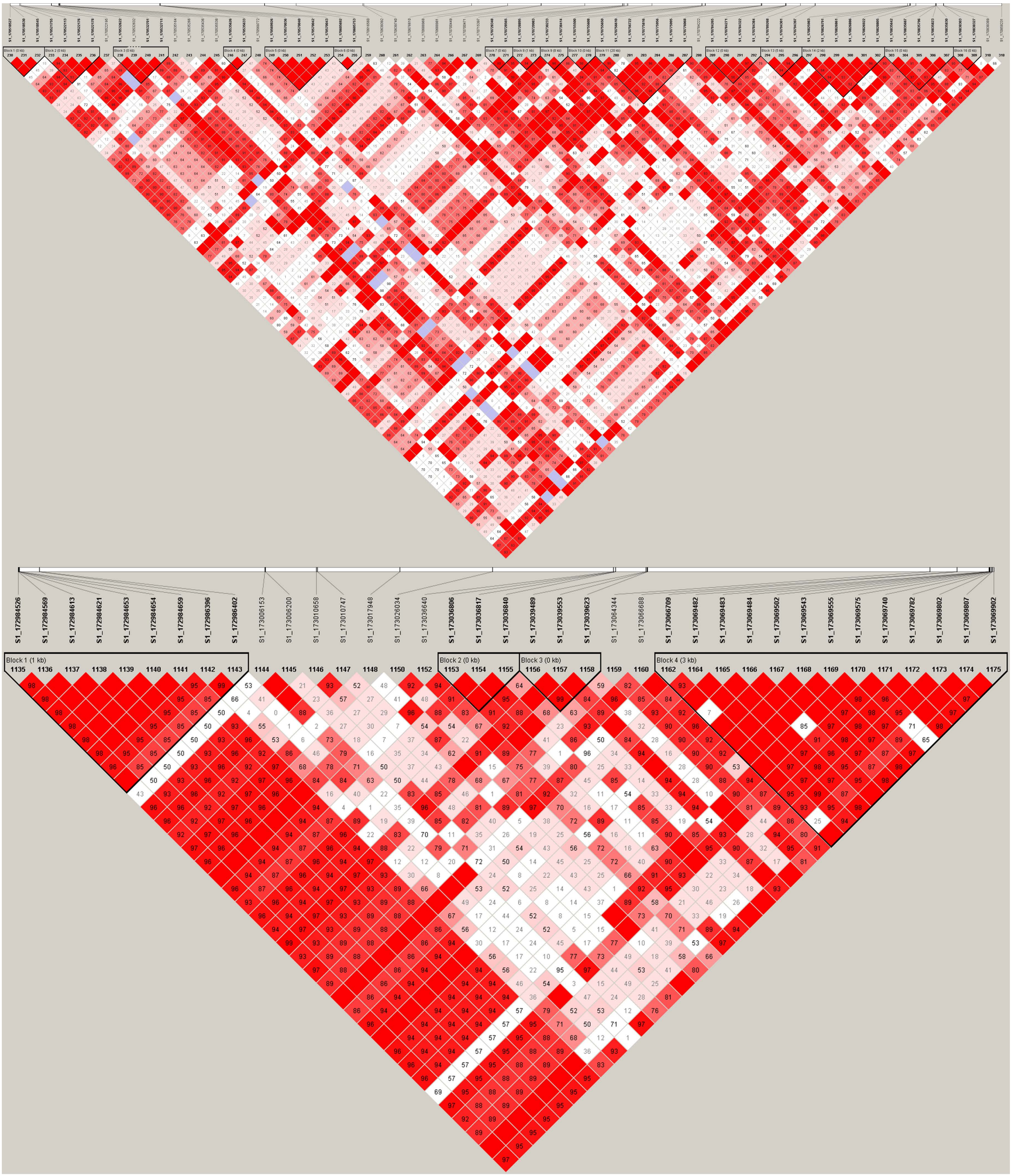

Fig S2. Mosaic pattern of two blocks. All SNPs measured by ddGBS in these two blocks of Fig 6 were used to construct the haplotype. The upper diagram is the haplotype of 73 SNPs located at 170.52 - 170.86 Mb, and the following diagram is the haplotype of 37 SNPs located at 172.98 - 173.07 Mb. The 9 SNPs from S1_172 984 526 to S1_172 986 402 were highly linked with all other SNPs.

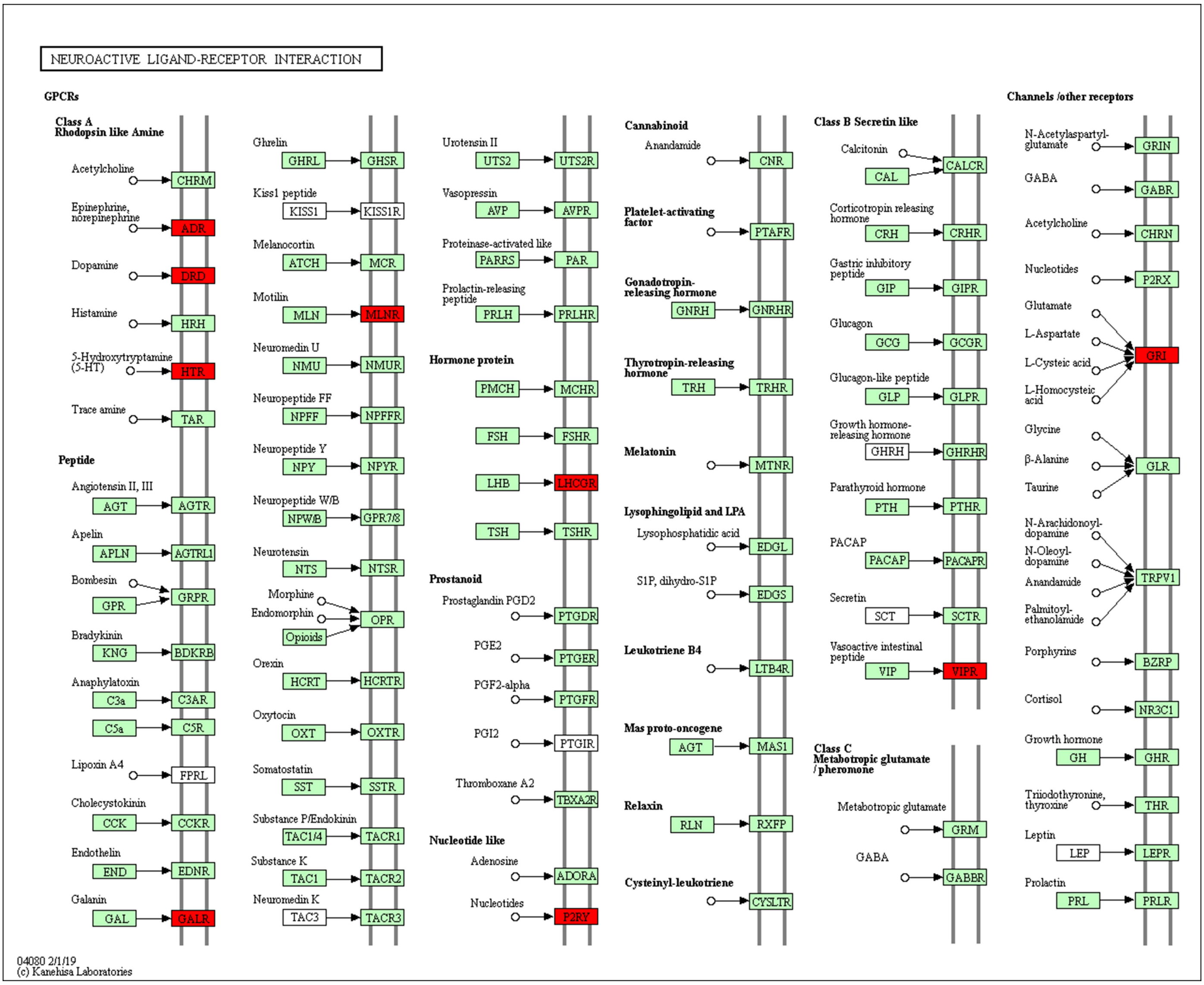

Fig S3. The neuroactive ligand-receptor interaction pathway was enriched by candidate genes. The genes with red bottom plates were the 10 candidate genes enriched in the neuroactive ligand-receptor interaction pathway, in which "VIPR" represents both ENSGALG00000005259 and ENSGALG00000006575.
